# β-Cyclodextrin/Isopentyl Caffeate Inclusion Complex: Synthesis, Characterization and Antileishmanial Activity

**DOI:** 10.3390/molecules25184181

**Published:** 2020-09-12

**Authors:** Carine S. F. Marques, Nathalia S. Barreto, Simone S. C. de Oliveira, André L. S. Santos, Marta H. Branquinha, Damião P. de Sousa, Mayara Castro, Luciana N. Andrade, Matheus M. Pereira, Classius F. da Silva, Marco V. Chaud, Sona Jain, Alini T. Fricks, Eliana B. Souto, Patricia Severino

**Affiliations:** 1 Postgraduation in Biotechnology Program, Industrial and Institute of Technology and Research (ITP), Tiradentes University (UNIT), Av. Murilo Dantas, 300, 49010-390 Aracaju, Brazil; carine.santaferreira@gmail.com (C.S.F.M.); nathaaliabarreto@hotmail.com (N.S.B.); sonajain24@yahoo.com (S.J.); alinitf@yahoo.com.br (A.T.F.); 2 Departament of General Microbiology, Institute of Microbiology Paulo de Góes, Federal University l Rio de Janeiro, 21941-918 Rio de Janeiro, RJ, Brazil; simonesantiagorj@yahoo.com.br (S.S.C.d.O.); andre@micro.ufrj.br (A.L.S.S.); mbranquinha@micro.ufrj.br (M.H.B.); 3 Department of Pharmaceutical Sciences, Federal University of Paraíba, 58051-900 Paraíba, Brazil; damiao_desousa@yahoo.com.br (D.P.d.S.); mayaracastrodemorais@gmail.com (M.C.); 4 Department of Medicine, Federal University of Sergipe, CEP 49400-000 Lagarto, Sergipe, Brazil; luciana.nalone@hotmail.com; 5 CICECO-Aveiro Institute of Materials, Departamento f Chemistry, University of Aveiro, 3810-193 Aveiro, Portugal; matheus.pereira@ua.pt; 6 Department of Exact Sciences and Earth, Federal University of São Paulo (UNIFESP), 09972-270 Diadema CEP, Brazil; classiusferreira@yahoo.com.br; 7 Department of Technological and Environmental Processes, Sorocaba University (UNISO), Rod. Raposo Tavares, Km 92.5, 18023-000 Sorocaba, Brazil; marco.chaud@prof.uniso.br; 8 Department of Pharmaceutical Technology, Faculty of Pharmacy, University of Coimbra, Pólo das Ciênciasda Saúde, Azinhaga de Santa Comba, 3000-548 Coimbra, Portugal; 9 CEB-Centre of Biological Engineering, University of Minho, Campus de Gualtar, 4710-057 Braga, Portugal; 10 Center for Biomedical Engineering, Department of Medicine, Brigham and Women′s Hospital, Harvard Medical School, 65 Landsdowne Street, Cambridge, MA 02139, USA; 11 Tiradentes Institute, 150 Mt Vernon St, Dorchester, MA 02125, USA

**Keywords:** isopentyl caffeate, β-cyclodextrin, inclusion complex, leishmania

## Abstract

Isopentyl caffeate (ICaf) is a bioactive ester widely distributed in nature. Our patented work has shown promising results of this molecule against Leishmania. However, ICaf shows poor solubility, which limits its usage in clinical settings. In this work, we have proposed the development of an inclusion complex of ICaf in β-cyclodextrin (β-CD), with the aim to improve the drug solubility, and thus, its bioavailability. The inclusion complex (ICaf:β-CD) was developed applying three distinct methods, i.e., physical mixture (PM), kneading (KN) or co-evaporation (CO) in different molar proportions (0.25:1, 1:1 and 2:1). Characterization of the complexes was carried out by thermal analysis, Fourier-transform infrared spectroscopy (FT-IR), scanning electron microscopy (SEM) and molecular docking. The ICaf:β-CD complex in a molar ratio of 1:1 obtained by CO showed the best complexation and, therefore, was selected for further analysis. Solubility assay showed a marked improvement in the ICaf:β-CD (CO, 1:1) solubility profile when compared to the pure ICaf compound. Cell proliferation assay using ICaf:β-CD complex showed an IC_50_ of 3.8 and 2.7 µg/mL against *L. amazonesis* and *L. chagasi* promastigotes, respectively. These results demonstrate the great potential of the inclusion complex to improve the treatment options for visceral and cutaneous leishmaniases.

## 1. Introduction

Leishmaniasis is a parasitic disease caused by protozoa of the genus Leishmania, showing two basic life cycle stages: an extracellular stage (promastigotes) found in the invertebrate host and an intracellular stage (amastigotes) found in the vertebrate host. Leishmania is transmitted to a mammalian host through the bite of sandflies belonging to the *Lutzomyia* and *Phlebotomine* genera, which feed on blood and inject the promastigote forms. In the vertebrate host, the parasites infect phagocytic cells, such as neutrophils, monocytes and mainly macrophages, where they differentiate into amastigote form. Amastigotes multiply and spread the infection, reaching different parts of the body, such as the liver, bone marrow, spleen and lymph nodes. Among different clinical forms of the disease, cutaneous (CL), and visceral (VL) leishmaniases are the most common [1].

Leishmaniasis is an emerging neglected tropical disease, endemic in approximately 98 countries worldwide. The disease is associated with factors, such as malnutrition, weakened immune systems and a lack of financial resources [2]. Approximately, 0.7 to one million new cases are reported annually worldwide, and close to one billion people live in endemic areas at the risk of infection [3]. According to the Drugs for Neglected Diseases initiative (DNDi), among the newly reported cases of leishmaniasis, around 0.6–1.2 million cases belong to CL and 50–90 thousand cases to VL, with 20–30 thousand cases evolving to death.

Conventional treatment options (e.g., amphotericin B, pentamidine isethionate and N-methyl glucamine antimoniate) suffer from side effects ranging from nephrotoxicity to cardiotoxicity and need strong patient compliance to prevent the risk of drug resistance and overcome therapeutic failures [4]. According to the World Health Organization (WHO), the control of leishmaniasis is a challenge, and the development of the new antileishmanial drugs to act on parasite targets is required for the improvement of treatment options [5].

**Figure 1:**
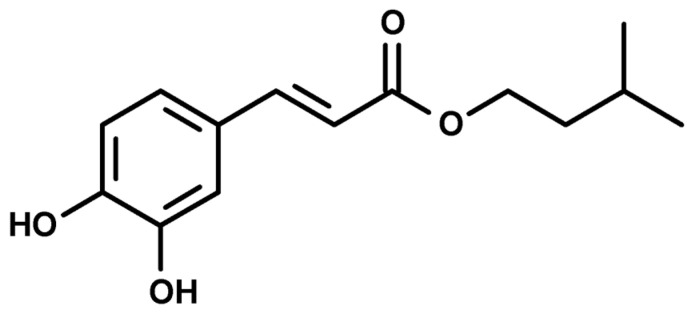
Chemical structure of isopentyl caffeate.

In this context, many studies report the bioactivity of derivatives of caffeic acid confirming its anti-inflammatory [6], antineoplastic [7], antiviral action, including anti-HIV [8], neuroprotective [9], anti-atherosclerotic [10], antioxidant [11] and antimicrobial properties [12]. Isopentyl caffeate (ICaf) is a bioactive organic ester (Figure 1), derived from caffeic acid and characterized as a gray crystalline solid with a molar mass of 250.29 g/mol [13]. In addition, ICaf is widely distributed in nature with promising results of this molecule against Leishmania in our patented work [14].

Our research group has been studying the effect of ICaf in both the promastigote and amastigote forms of cutaneous (*L. amazonensis*) and visceral (*L. chagasi*) leishmaniases [14]. However, the low solubility of ICaf due to its hydrophobic character is a great challenge which limits its usage in clinical settings. In this work, we have proposed the development of an inclusion complex of ICaf in β-cyclodextrin (β-CD), with the aim to improve the drug solubility, and thus, its bioavailability.

Cyclodextrins (composed of d-glucopyranose units) are functional pharmaceutical excipients that have gained widespread attention due to their ability to interact with poorly water-soluble drug candidates, thereby improving their solubility [15]. Their conical spatial structure and the orientation of the hydroxy groups on the outer shell gives these cyclic sugars unique physicochemical properties, capable of being solubilized in an aqueous medium and at the same time encapsulating hydrophobic molecules in their internal cavity space [16]. Cyclodextrin has been widely used for controlled drug release and is reported to improve the dissolution rate, with a possible increase in bioavailability [17,18]. The present study aimed to develop and characterize ICaf:β-CD inclusion complexes by different methodologies. Subsequently, the effects of ICaf:β-CD on the in vitro proliferation of *L. amazonensis* (agent of cutaneous leishmaniasis) and *L. chagasi* (agent of visceral leishmaniasis) were evaluated as a proof of formulation efficiency.

## 2. Results and Discussion

Thermal analysis is a crucial tool to suggest the formation of inclusion complexes [19]. We have used Differential Scanning Calorimetry (DSC) and Thermogravimetry/Derivative Thermogravimetry (TG/DTG) to study the physical properties of the inclusion complexes as a function of temperature and time. The complex is formed when the guest molecule, usually in crystalline form, represented by a narrow peak on the DSC curve, decreases or disappears due to the loss of the crystalline structure caused by complexation [20]. TG/DTG curves indicate the thermal stability of the samples by change/loss in their mass, as a function of temperature [21].

DSC profiles of the pure components (ICaf and β-CD) and complexes (ICaf:β-CD) are shown in Figure 1. The DSC curve of ICaf showed an endothermic event between 127.63 and 133.77 °C (Table 1), corresponding to its melting peak. The β-CD showed two endothermic events between 63.80–142.16 °C, and 308.71–343.18 °C. The first endothermic event is related to the loss of water and the second event is related to its melting peak [19], similar to results obtained by other studies [18,22,23,24].

**Figure 2:**
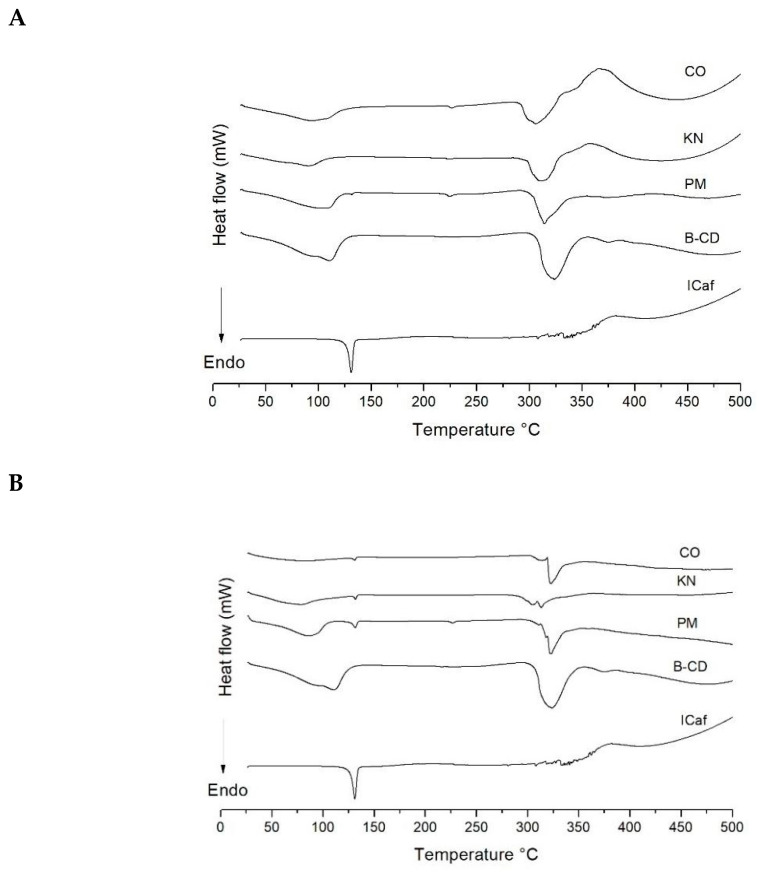

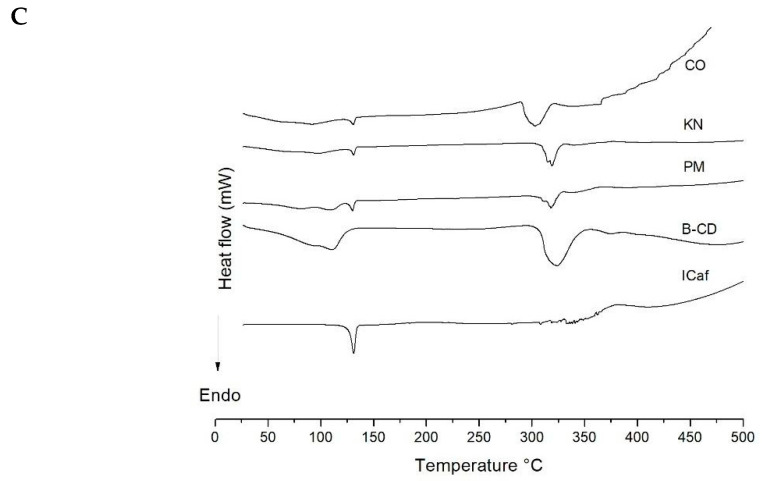
Differential scanning calorimetry curves of isopentyl caffeate (ICaf), β-cyclodextrin (β-CD), and inclusion complex (ICaf:β-CD) prepared by physical mixture (PM), kneading (KN), co-evaporation (CO) using molar ratios of (**A**) 0.25:1, (**B**) 1:1 and (**C**) 2:1.

The CO (ICaf:β-CD, 1:1) thermogram suggests that this method offers a more efficient complexation, attributed to the presence of water favoring the incorporation of ICaf in the internal cavity of β-CD. The melting peak of ICaf (127.63–133.77 °C) showed a reduction in its peak. Additionally, a decreased endothermic event for the β-CD between 63.80–142.16 °C can be observed (Figure 2C). During complexation, the water molecules present in the β-CD are removed and replaced by the guest molecule, favoring the formation of the complex, and changing the thermoanalytical curve of the systems. Thus, complexation contributes to the stability of the guest molecule [25]. For the PM and KN methods, the reduction in the melting peak was smaller compared to CO and it is also observed that the water loss event of β-CD between 63.80–142.16 °C did not disappear when compared to the same event in the CO method.

**Figure 3:**
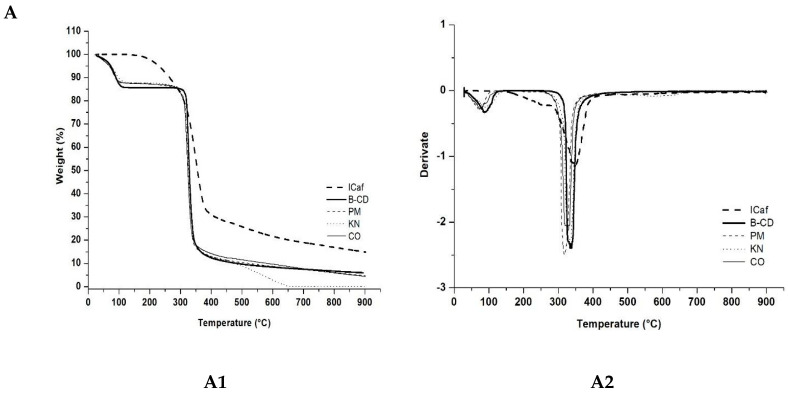

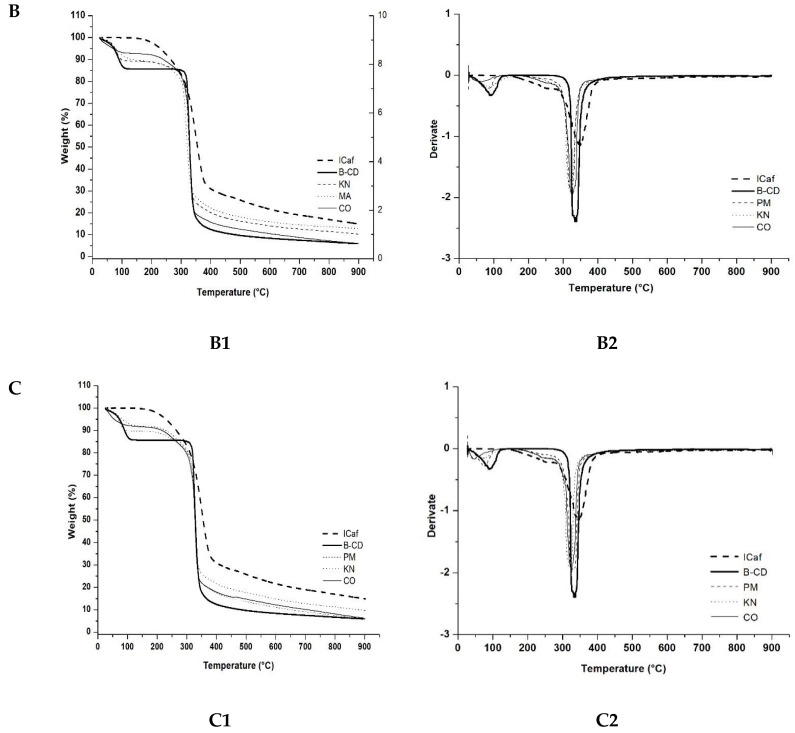
Thermogravimetric (A1, B1, C1) and derivative thermogravimetric (A2, B2, C2) analyses of isopentyl caffeate (ICaf), β-cyclodextrin β-CD, and inclusion complex prepared by physical mixture (PM), kneading (KN) and co-evaporation (CO) using molar ratios of (ICaf: β-CD) of (**A**) 0.25:1, (**B**) 1:1 and (**C**) 2:1.

Figure 3 shows the TG/DTG curves and Table 2 shows mass loss calculated from specific intervals for each sample. The TG/DTG curves of ICaf revealed a significant mass loss (68.33% ∆m1.2) starting at 170 °C and ending at approximately 400 °C. The loss of mass occurs gradually. The TG/DTG curves of β-CD showed a mass loss event between 30–170 °C (13.57% ∆m1) attributed to the loss of water molecules from the β-CD cavity, and another event with a significant and rapid mass loss between 280–400 °C (73.15% ∆m3) due to the degradation of β-CD. Similar mass loss events for β-CD were reported previously [20,23,26].

For CO method (1:1 ratio, Figure 3B), a decrease in the peak related to the β-CD water loss event can be observed, suggesting an improved complexation by replacing the water molecule in β-CD by ICaf. There is also a change in the profile of the fusion peak of β-CD with the start of decomposition of the material at a temperature of 170° C suggesting the presence of the ICaf molecule inside the β-CD. The β-CD decomposition event started only at approximately 280° C. The thermogravimetric profiles of the complexes using the PM and KN methods (Figure 3) in a 1:1 ratio did not show a significant reduction in the β-CD dehydration temperature range, suggesting that the complexation did not occur effectively. The thermogravimetric profiles of ICaf:β-CD in different proportions (0.25:1, 1:1, 2:1) and methods (PM, KN and CO) revealed changes in endothermic events in comparison with pure substances (Figure 3). However, the most significant difference was found in the CO method (1:1) between 30–170 °C with a reduction in the peak of the thermogravimetric curve (Figure 3B). According to Galvão et al. [19], the replacement of the water molecule found in the internal cavity of the β-CD generates an equilibrium of dynamic complexation.

FTIR analyses allow the identification of functional groups existing in the inclusion complexes, through the identification of vibrational and rotational movements of molecular bonds, and it is one of the experimental methods that was used to observe intermolecular interactions between ICaf and CDs [22]. The formation of inclusion complexes between a drug and β-CD is a process that involves chemical interaction through hydrogen bonding due to the hydrophobic interactions of the internal cavity of the β-CD and the active compound. Such interactions can be observed by changes (increase or decrease) in one or more frequencies of the infrared spectrum [27].

**Figure 4:**
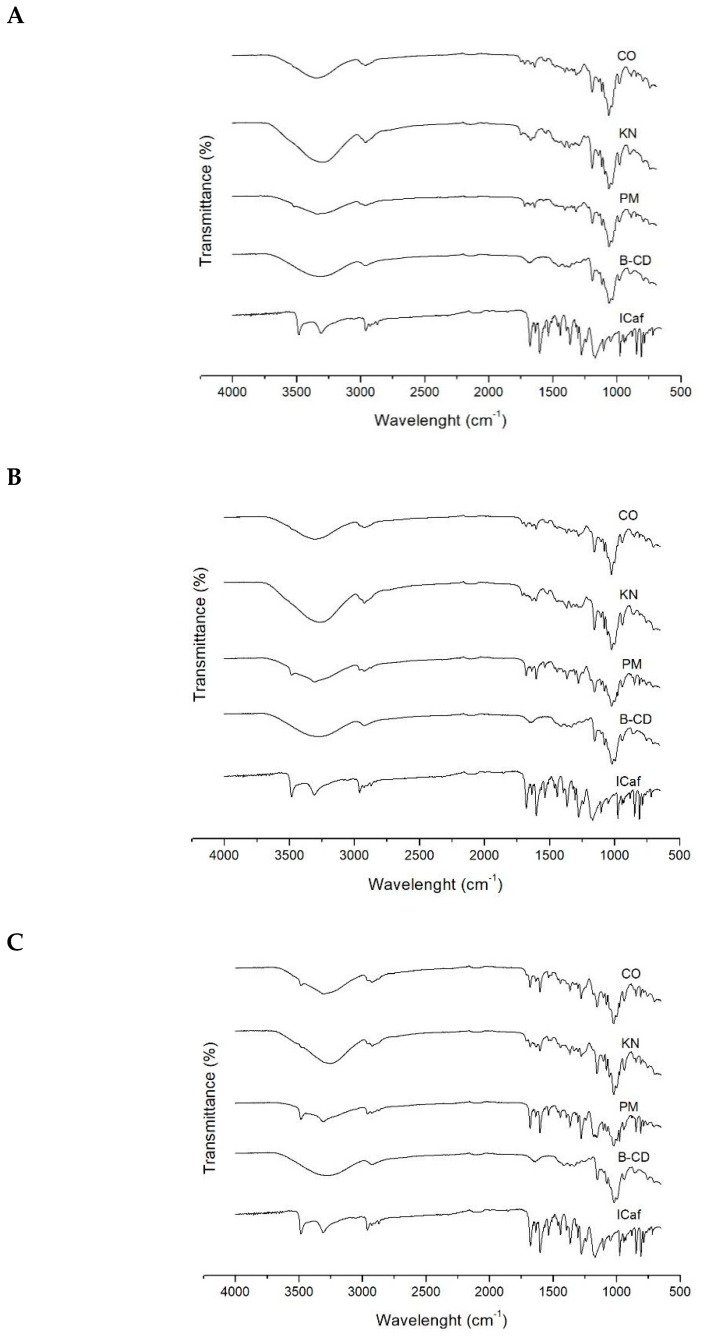
Fourier transform infrared spectroscopy spectra of isopentyl caffeate (ICaf), β-cyclodextrin (β-CD), and inclusion complex prepared by physical mixture (PM), kneading (KN) and co-evaporation (CO) using molar ratios of (ICaf:β-CD) of (**A**) 0.25:1, (**B**) 1:1 and (**C**) 2:1.

In Figure 4, differences were observed in the FT-IR spectra for the ICaf:β-CD complex compared to the pure substances. The ICaf spectra appeared as a weak band between 3500 cm^−1^–3520 cm^−1^ due to the presence of the hydroxyl group. The absorption band of the C-H group present in aromatic compounds could be identified between 3000 cm^−1^ and 3130 cm^−1^. The presence of carbonyl (C=O) in the ester group was observed between 1750–1740 cm^−1^. The bonds between the carbons (C=C) of the ICaf aromatic ring appeared between 1600–1450 cm^−1^ and the C-O bonds appeared between 1300–1250 cm^−1^ and 1200–1050 cm^−1^. The presence of (HC=CH) group was observed between 970–960 cm^−1^. It was also possible to observe the presence of the aromatic ring between 810–750 cm^−1^. Araújo et al. (2019) have reported similar results for ICaf between 1600 and 1450 cm^−1^ due to the presence of the C=C bond of the aromatic ring.

The β-CD spectrum showed a strong and wideband between 3600–3000 cm^−1^, due to the presence of O-H functional group that is responsible for the hydrophilic characteristic of β-CD. Presence of C-H functional group of the cyclic structure can be observed between 2960 cm^−1^ and 2850 cm^−1^. A low-intensity band due to a carbon–carbon double bond (C=C) could be observed between 1675 and 1645 cm^−1^ due to the presence of a double bond between the carbon atoms. The simple bonds between carbon and oxygen (C-O) were observed between 1200–1050 cm^−1^. Similar results were reported elsewhere [17,28].

The inclusion complexes obtained by the CO method in the proportions 0.25:1 and 1:1 were the most suggestive of complexation, as a decrease in the intensity of the ICaf absorption band was seen between 3500–3520 cm^−1^ attributed to the hydroxyl group, which is broken to perform hydrogen bonding with the host molecule. The absorption spectrum of β-CD between 3600–3000 cm^−1^ also reduced its intensity, becoming a weaker band. This occurred possibly due to the replacement of O-H group in the internal cavity the β-CD, by the ICaf molecule.

In PM method, there was no evidence of complex formation, as the spectrum of pure ICaf between 3500–3520 cm^−1^ was kept, indicating that the ICaf molecule was not incorporated in the β-CD cavity. For the KN method, no complex formation was suggested as the band resulting from the presence of the β-CD water molecule continued to be prominent.

**Figure 5:**
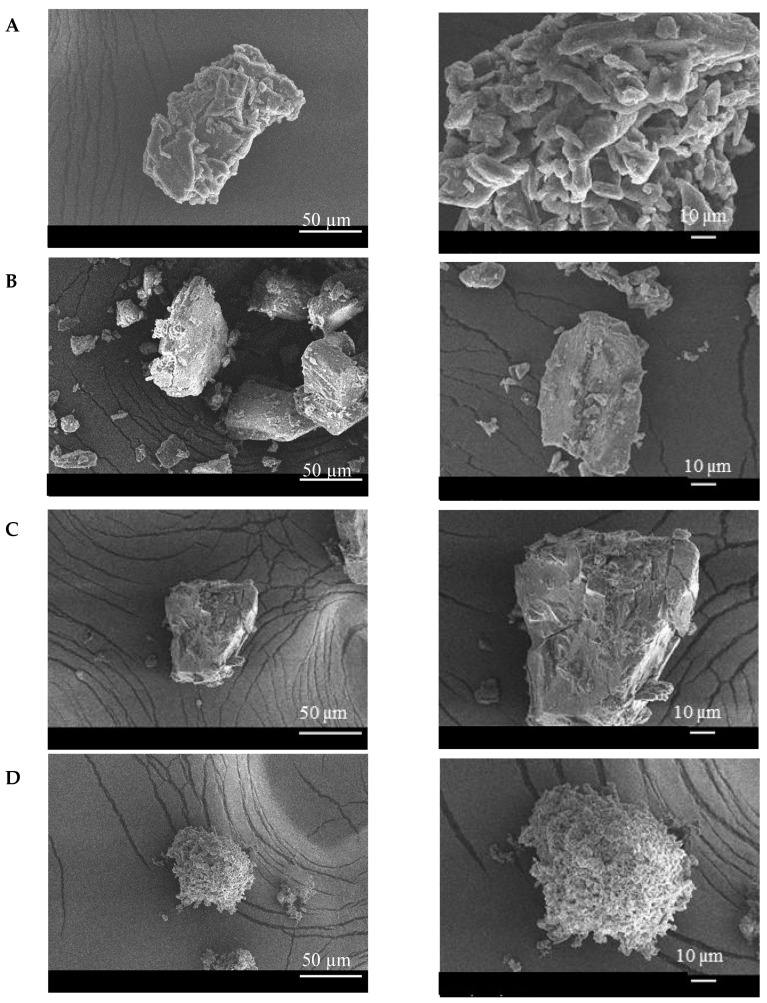
Scanning electron microscopy micrographs of isopentyl caffeate (ICaf) (**A**), β-cyclodextrin (β-CD) (**B**) and inclusion complex (ICaf:β-CD) prepared by physical mixture (PM) (**C**) and co-evaporation (CO) (**D**) using 1:1 molar ratios. **Left:** ×500 magnification, **Right:** ×1000 magnification.

SEM is a technique widely used to qualitatively define the formation of inclusion complexes. Figure 5 shows the SEM micrographs of ICaf, β-CD, ICaf:β-CD (1:1) obtained by CO and PM methods. Microscopic pictures clearly show a compact and well-defined structure of ICaf with a three-dimensional appearance. The β-CD presents crystalline particles of irregular size present in the form of parallelograms, with a distinct surface and contour. ICaf:β-CD obtained by PM did not change significantly compared to pure substances (β-CD and ICaf). This observation suggests that there is no interaction between them, since each component is present with its original morphology [29]. The inclusion complex obtained by the CO method showed significant changes in the shape and size of the particles, resulting in compact and homogeneous agglomerates that indicate a modification of the three-dimensional structure of the ICaf. The microscopic structure of the ICaf showed an irregular surface, without well-defined edges. The β-CD showed a smooth surface, shaped like a parallelogram in different sizes with well-defined ends. The inclusion complex formed by the CO (1:1) method, revealed an amorphous, granular aspect with a porous surface.

**Figure 6:**
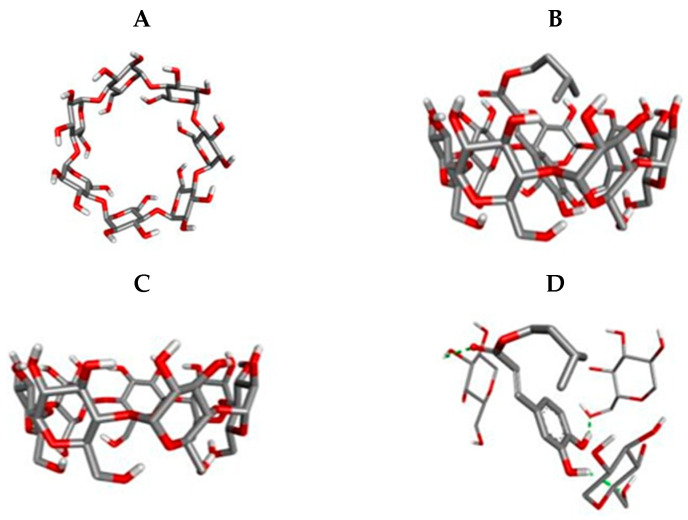
Molecular structure of β-CD (**A** and **C**), lowest docking energy conformation of ICaf with β-CD (**B**) and molecular interactions (H-bond) of ICaf with β-CD (**D**).

Molecular docking was used to predict possible molecular arrangements between ICaf and β-CD, type of bond involved and the spatial conformation of the inclusion complex. This analysis was carried out by testing different forms of molecular arrangements until an arrangement with the lowest energy value was obtained as it is most likely to justify the interaction [30,31]. The molecular structure of β-CD is shown in Figure 6A,C. Among the arrangement attempts, the ICaf molecular fit that showed the lowest absolute affinity value (kcal/mol) is shown in Figure 5B. To better identify, at the molecular level, the coupling of ICaf in the β-C structure, the diagram of molecular interactions is shown in Figure 5D.

According to the obtained results, it is suggested that the hydroxyl and oxygen groups present in ICaf are involved in hydrogen bonding with β-CD. Figure 6D showed the complexation of the ICaf in the β-CD cavity through the hydroxyl and oxygen groups (marked in green dots). The best anchoring position and affinities, interacting molecules, type of interaction, and geometry distance (Å) are shown in Table 3.

The smallest geometric distance (less than 4 Å) between ICaf atoms contributes to stabilization in the β-CD cavity since the potential for intermolecular interaction declines with the increase in the distance between the particles [22]. The result of the molecular coupling corroborates the FT-IR spectra, confirming the involvement of intermolecular hydrogen bonds [32]. Additionally, molecular docking confirmed that inclusion complexes in a 1:1 stoichiometry ratio are most stable.

**Figure 7:**
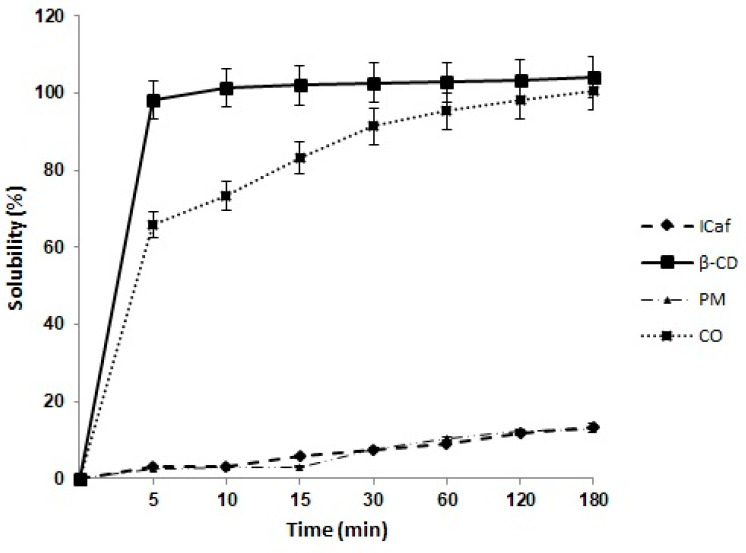
Comparison of the solubility profiles of Isopentyl Caffeate (ICaf), β-cyclodextrin (β-CD) and inclusion complex (ICaf:β-CD) prepared by physical mixture (PM) and co-evaporation (CO) in 1:1 molar ratios (*n* = 3).

The solubility of ICaf, β-CD and ICaf:β-CD (PM, 1:1 and CO, 1:1) was evaluated after determining the concentration of the compounds in the dissolution medium. The results obtained are expressed as a percentage of drug released versus time in Figure 7. The pure ICaf and PM (1:1) presented a low dissolution profile, probably due to its hydrophobic character. Only with the use of β-CD it was possible to observe an increase in the percentage of ICaf dissolved in the medium in relation to pure ICaf, reaching approximately 70% of release in the initial 5 min. The passage of the drug from the crystalline form to the amorphous state is a factor that generally makes the systems more soluble, presenting a higher dissolution rate [24]. The change in the morphology of ICaf when complexed ICaf:β-CD (1:1) was already evidenced in the SEM micrographs discussed above.

**Figure 8:**
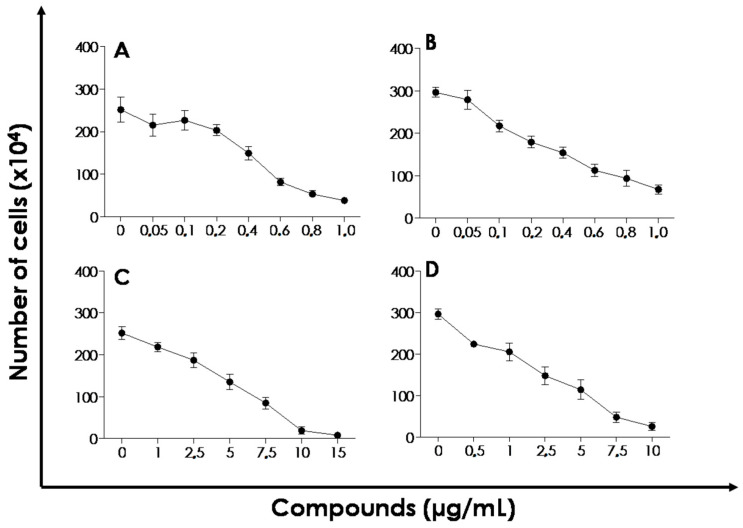
The effect of ICaf on the proliferation of *L. amazonensis* (**A**) and *L. chagasi* (**B**). Effects of the inclusion complex produced by co-evaporation (ICaf:β-CD) on the proliferation of *L. amazonensis* (**C**) and *L. chagasi* (**D**) promastigote forms in different concentrations. Viable cells were counted after 72 h using Neubauer chamber. Data shown represent the mean ± standard error of three independent experiments performed in triplicate.

As shown in Figure 8, ICaf:β-CD (Figure 8C,D) inhibited the growth of *L. amazonensis* and *L. chagasi* promastigotes in a typical dose-dependent manner. After 72 h of incubation, the IC_50_ values of ICaf:β-CD calculated for *L. amazonensis* (Figure 8C) and *L. chagasi* (Figure 8D) were 3.8 and 2.7 µg/mL, respectively, suggesting that the promastigote forms are susceptible to the action of ICaf:β-CD. ICaf alone showed IC_50_ value of 0.39 µg/mL and 0.43 µg/mL for *L. amazonensis* (Figure 8A) and *L. chagasi* (Figure 8B), respectively. No post-test was needed to compare the evaluated compounds, since the recorded IC_50_ data (concentration-response curve) allow us to select which compounds have greater pharmacological potency and consequently improved outcomes. However, it is important to note that the amount of ICaf (in mg) present in the complex (equimolar ratio 1:1) is lesser when compared to the pure ICaf used in the proliferation assay. As expected, miltefosine, which is a well-known antileishmanial drug, was able to reduce the in vitro proliferation of both Leishmania species (Table 4). Ceole et al. (2017) and da Câmara Rocha et al. (2019) [33] classified the antileishmanial activity of compounds based on the IC_50_ values: 10 µg/mL—high activity; 10–50 µg/mL—considerate activity; 50–100 µg/mL—moderate activity, and 100 µg/mL—no activity. Thus, inclusion of ICaf in β-CD could increase its solubility at the same time maintaining high antileishmanial activity. β-CD alone did not show antileishmanial activity. As mentioned before, β-CD has been widely used for controlled drug release. It is reported to improve the dissolution rate, with a possible increase in bioavailability, thus, also reducing the drug dosage [17,34]. There is also the possibility of reducing gastric irritation, unpleasant taste and odor (when used orally), and reduction in dermal irritation when using topically [35,36]. Further in vitro and in vivo studies are currently in progress to explore the pharmaceutical potential of the ICaf:β-CD complex for both oral and topical usage against visceral and cutaneous leishmaniasis.

## 3. Materials and Methods

### 3.1. Materials

ICaf was synthesized according to the methodology described by Araujo et al. [6]. All other reagents, including β-CD (seven glucose units), were purchased from Sigma-Aldrich (St. Louis, MO, USA). Ultra-pure water was used for all the experiments carried out in this study.

### 3.2. ICaf:β-CD Inclusion Complexes

The formation of (ICaf:β-CD) complex was performed by physical mixture (PM), co-evaporation (CO) and kneading (KN) methods using 0.25:1, 1:1 and 2:1 molar ratios. The PM method consisted of light homogenization of both the components with a mortar. The CO technique utilized manual mixing of the components followed by progressive addition of distilled water (400 µL) forming a solution, while for preparing the inclusion complex by KN method, β-CD and ICaf were kneaded and dispersed in 8 mL of water using magnetic stirring (Kasvi, K40-1810, Kasvi São Paulo, Brazil) for 36 h to obtain a paste. All samples were stored in hermetically sealed containers [37].

### 3.3. Thermal Analysis

Differential scanning calorimetry (DSC) curves (DSC 2010 TA Instruments, Mettler Toledo, Columbus, OH, USA) were generated using a temperature range of 25–500 °C, under a nitrogen (N2) atmosphere, a 50 mL min^−1^ gas flow rate and a 10 °C min^−1^ heating ratio. The sample holder made of aluminum, contained 2 mg of the sample. Thermogravimetric (TG/DTG, Mettler Toledo, Columbus, OH, USA) curves were obtained on Shimadzu TG-60 (Kyoto, Japan) analyzer with a temperature ranging from 25 to 900 °C, under a (N2) atmosphere, a gas flow rate of 50 mL min^−1^ and a 10 °C min^−1^ heating ratio. The analyzes were performed using a platinum sample holder containing 7 mg of the sample [38].

### 3.4. Fourier-Transform Infra-Red Analysis (FTIR)

FTIR spectroscopy was carried out using Agilent Caryn 630 FTIR (Agilent Technologies, Wilmington, DE, USA) with attenuated total reflectance device (Miracle ATR, Pike Technologies Spectroscopic Creativity, Madison, WI, USA), and selenium crystal. The spectra were obtained between 400–4000 cm^−1^ with resolution 2 cm^−1^ and processed for automatic data acquisition by Agilent Microlab PC software (Santa Clara, CA, USA) [39].

### 3.5. Scanning Electron Microscopy (SEM)

The morphological characteristics of the ICaf, β-CD, and ICaf:β-CD were analyzed by SEM (Hitachi TM 3000, Tokyo, Japan). The samples were mounted on aluminum stubs, subjected to gold beam metallization and analyzed using an 8 kV voltage acceleration electron microscope [39]. Only the inclusion complexes (ICaf:β-CD) developed by the methods of physical mixture (PM) and co-evaporation (CO) in the molar ratio of 1:1 were characterized by SEM.

### 3.6. Molecular Docking

The interactions of β-CD and ICaf were identified using the Auto-dock vina 1.1.2 program [40]. The molecular structure of β-CD was created through Discovery Studio, v 20 (Accelrys, San Diego, CA, USA) and used in molecular docking. Auto DockTools (ADT) [41] was used to prepare the β-CD input file by merging non-polar hydrogen atoms, adding partial charges and atom types. ICaf atomic coordinates were computed by a DS Visualizer and ligand rigid root was generated using AutoDockTools (ADT), setting all possible rotatable bonds defined as active by torsions. The grid center at the center of mass for β-CD was −26.173 Å × −30.009 Å × −13.283 Å. in the x-, y-, and z-axes, respectively. The grid dimension was 40 Å × 40 Å × 40 Å to cover the whole interaction β-CD surface. The binding model with lowest binding free energy was searched out from 10 different conformers.

### 3.7. Dissolution Assay

The dissolution assay of ICaf, β-CD and ICaf:β-CD was performed to investigate the influence of β-CD on the solubilization rate of ICaf. A known amount of ICaf corresponding to 10 mg was added to 40 mL of phosphate buffer solution (pH 7.5) as a dissolution medium in sealed flasks and subsequently subjected to a hot bath maintained at 37 ± 1°C, and a stirring rate of 20 rpm. Aliquots (5 mL) were collected in 5, 10, 15, 30, 60, 120 and 180 min and filtered through a 0.45 µm pore membrane. The quantification of the dissolved drug was performed by means of absorption spectrophotometry in the ultraviolet region, at 290 nm, using a Shimadzu spectrophotometer (UV-2600 Shimadzu Tokyo, Japan). The amount of drug release into the dissolution medium was determined as a function of time [24].

### 3.8. Biological Assays

#### Effect of ICaf:β-CD on the Proliferation of L. amazonensis and L. chagasi

The effect of ICaf:β-CD complex on the proliferation rate was assessed using promastigote form of both L. amazonensis and L. chagasi. Briefly, the promastigote cells were quantified using a Neubauer chamber and re-suspended in fresh medium. A final concentration of 5 × 105 viable promastigotes/mL was utilized. The ICaf:β-CD was used at final concentrations of 1–15 µg/mL for L. amazonensis, 0.5–10 µg/mL for L. chagasi. ICaf was used at 0.05–1 µg/mL concentration. The parasites were incubated at 28 °C and the number of viable promastigotes was quantified by counting the flagellates in a Neubauer chamber. The IC50, a value that refers to the concentration of the drug that causes a 50% reduction in parasite proliferation compared to the control growth curve, was calculated after 72 h of incubation through linear regression of the log of the number of viable cells versus the drug concentration using the Excel program (2010, Microsoft, Redmond, Washington, WA, USA) [42]. Miltefosine, a classical anti-Leishmania drug, was used for comparison purpose [43,44]. The compounds (ICaf, β-CD and ICaf:β-CD) were dissolved in 5% dimethyl sulfoxide (DMSO) for IC50 measurements.

### 3.9. Statistical Analysis

The experiments were performed in triplicate, as three independent experiments. Data are presented as mean ± SEM (or SD) or IC_50_ values, and their 95% confidence intervals (CI 95%) were obtained by nonlinear regression. All analyses were carried out using the Graphpad program (Intuitive Software for Science, San Diego, CA, USA) and Microsoft Excel 2010 (2010, Microsoft, Redmond, Washington, WA, USA).

## 4. Conclusions

ICaf, a bioactive ester with anti-leishmania action, was complexed with β-cyclodextrin by different methods and physicochemically characterized by different methodologies. Our study showed that ICaf: β-CD forms the best inclusion complexes with β-CD in the stoichiometric ratio of 1:1, produced by the CO method. The complex showed greater solubility compared to ICaf alone and may be a promising alternative to improve the treatment of visceral and cutaneous leishmaniasis as it compromised the in vitro proliferation of *L. amazonensis* and *L. chagasi*.

## Figures and Tables

**Table 1:** Thermal properties of isopentyl caffeate (ICaf), β-cyclodextrin (β-CD), and inclusion complex (ICaf:β-CD) prepared by physical mixture (PM), kneading (KN), co-evaporation (CO) using molar ratios of (A) 0.25:1, (B) 1:1 and (C) 2:1.

Sample	Events	T_onset_ (°C)	T_endset_ (°C)	∆H (J)
β-CD	1	63.80	142.16	1.73
	2	308.71	343.18	1.53
ICaf	1	127.63	133.77	0.22
PM 0.25:1	1	61.92	118.56	0.26
	2	128.80	133.67	0.01
	3	220.79	228.54	0.02
	4	302.49	328.33	0.89
KN 0.25:1	1	96.04	106.75	0.32
	2	298.69	326.48	1.10
CO 0.25:1	1	72.01	124.52	0.71
	2	223.29	230.85	0.02
	3	292.41	327.90	1.56
PM 1:1	1	64.30	103.73	0.76
	2	127.67	134.52	0.05
	3	319.40	332.30	0.51
KN 1:1	1	43.22	93.39	0.58
	2	130.14	133.54	0.01
	3	309.49	320.60	0.48
CO 1:1	1	129.19	132.79	0.01
	2	318.53	320.40	0.51
PM 2:1	1	128.11	132.68	0.09
	2	313.51	325.19	0.27
KN 2:1	1	130.08	133.17	0.04
	2	311.19	326.05	0.38
CO 2:1	1	126.87	133.22	0.04
	2	291.04	322.76	0.64

**Table 2:** Mass loss obtained by TG of isopentyl caffeate (ICaf), β-cyclodextrin (β-CD), and inclusion complex prepared by physical mixture (PM), kneading (KN) and co-evaporation (CO) using molar ratios of (ICaf:β-CD) of 0.25:1, 1:1 and 2:1.

Sample	∆_m1_ (%) 30–170 °C	∆_m2_ (%) 170–280 °C	∆_m3_ (%) 280–400 °C	∆_m4_ (%) 400–900 °C
ICaf	0.72	11.99	56.34	16.05
β-CD	13.57	0.09	73.15	13.19
PM 0.25:1	12.40	1.93	72.68	8.34
KN 0.25:1	11.72	1.43	73.29	12.87
CO 0.25:1	12.00	1.29	71.83	9.81
PM 1:1	10.9	3.74	65.28	9.85
KN 1:1	10.62	5.58	61.61	9.47
CO 1:1	6.48	6.49	70.31	9.74
PM 2:1	9.81	6.05	66.04	11.85
KN 2:1	8.26	7.23	62.85	11.99
CO 2:1	7.73	8.30	65.21	11.66

**Table 3:** Molecular docking affinity, energy and interaction groups calculated by AutoDockvinna for β-cyclodextrin and isopentyl caffeate inclusion complex.

Compound	Affinity (kcal/mol)	Atoms de Interaction	Type of Interaction	From	For	Distance (Å)
Isopentyl caffeate	−4.9	O	Hydrogen bond	β-CD	ICaf	2.51
		H				
				ICaf	β-CD	2222
		H				2.05
						2.15

**Table 4:** Effect of test compounds on the in vitro proliferation of *L. amazonensis* and *L. chagasi* promastigotes.

Compounds	IC_50_ (µM) *L. amazonensis*	IC_50_ (µM) *L. chagasi*
ICaf	1.56 (1.35–1.73)	1.71 (1.46–1.88)
ICaf:β-CD (CO, 1:1)	2.74 (2.33–2.91)	1.95 (1.64–2.11)
β-CD	88.10 (67.21–95.6)	88.10 (67.21–95.6)
Miltefosine	6.69 (5.74–8.04)	6.38 (4.85–7.94)

Data are presented as IC_50_ values (µM) and their 95% confidence interval obtained by non-linear regression from three independent experiments performed in triplicate, measured by viable cells after 72 h using a Neubauer chamber. Miltefosine was used as the positive control.

## References

[1] Elisama A.C., Silva R.d.S., Gabriela C.d.C., Aline G.M.F., Maria L.d.C.B., Andre L.S.d., Helena C.C., Viviane L. (2014). Leishmaniasis: History, evolution of treatment and the need for new drugs. J. Curr. Biotechnol..

[2] Souto E.B., Dias-Ferreira J., Craveiro S.A., Severino P., Sanchez-Lopez E., Garcia M.L., Silva A.M., Souto S.B., Mahant S. (2019). Therapeutic Interventions for Countering Leishmaniasis and Chagas′s Disease: From Traditional Sources to Nanotechnological Systems. Pathogens.

[3] Status of Endemicity of Visceral Leishmaniasis, Worldwide, 2018. https://www.who.int/leishmaniasis/burden/GHO_VL_2018.pdf.

[4] Abamor E.S. (2017). Antileishmanial activities of caffeic acid phenethyl ester loaded PLGA nanoparticles against Leishmania infantum promastigotes and amastigotes in vitro. Asian Pac. J. Trop. Med..

[5] Zulfiqar B., Shelper T.B., Avery V.M. (2017). Leishmaniasis drug discovery: Recent progress and challenges in assay development. Drug Discovery Today.

[6] Schröter D., Neugart S., Schreiner M., Grune T., Rohn S., Ott C. (2019). Amaranth′s 2-Caffeoylisocitric Acid—An Anti-Inflammatory Caffeic Acid Derivative That Impairs NF-κB Signaling in LPS-Challenged RAW 264.7 Macrophages. Nutrients.

[7] Henah M.B., Taseen G., Ehtishamul H. (2016). Anti-neoplastic and calcium modulatory action of caffeic acid phenethyl ester and dasatinib in C6 glial cells: A therapeutic perspective. CNS Neurol. Disorders-Drug Targets.

[8] Langland J., Jacobs B., Wagner C.E., Ruiz G., Cahill T.M. (2018). Antiviral activity of metal chelates of caffeic acid and similar compounds towards herpes simplex, VSV-Ebola pseudotyped and vaccinia viruses. Antiviral Res..

[9] Morroni F., Sita G., Graziosi A., Turrini E., Fimognari C., Tarozzi A., Hrelia P. (2018). Neuroprotective effect of caffeic acid phenethyl ester in a mouse model of Alzheimer′s disease involves Nrf2/HO-1 pathway. Aging Dis..

[10] Calabriso N., Scoditti E., Massaro M., Pellegrino M., Storelli C., Ingrosso I., Giovinazzo G., Carluccio M.A. (2016). Multiple anti-inflammatory and anti-atherosclerotic properties of red wine polyphenolic extracts: Differential role of hydroxycinnamic acids, flavonols and stilbenes on endothelial inflammatory gene expression. Eur. J. Nutr..

[11] Tolba M.F., Omar H.A., Azab S.S., Khalifa A.E., Abdel-Naim A.B., Abdel-Rahman S.Z. (2016). Caffeic acid phenethyl ester: A review of its antioxidant activity, protective effects against ischemia-reperfusion injury and drug adverse reactions. Crit. Rev. Food Sci. Nutr..

[12] Arasoğlu T., Derman S. (2018). Assessment of the Antigenotoxic Activity of Poly (d, l-lactic-co-glycolic acid) Nanoparticles Loaded with Caffeic Acid Phenethyl Ester Using the Ames Salmonella/Microsome Assay. J. Agric. Food Chem..

[13] Araújo M.O., Freire Pessoa H.L., Lira A.B., Castillo Y.P., de Sousa D.P. (2019). Synthesis, Antibacterial Evaluation, and QSAR of Caffeic Acid Derivatives. J. Chem..

[14] Marques C.S.F., Severino P., Fricks A.T., dos Santos A.L.S., Andrade L.N., Chaud M., Souto E.B., Pergentinho D., de Oliveira S.S.C. (2020). Processo de Obtenção de Complexo de Inclusão de Cafeato de Isopentila em Ciclodextrina e Produto Obtido Para o Tratamento da Leishmaniose. Patent.

[15] Angelina A., Catherine R.-L., Adam B. (1999). Drug–Cyclodextrin Association Constants Determined by Surface Tension and Surface Pressure Measurements: I. Host—Guest Complexation of Water Soluble Drugs by Cyclodextrins: Polymyxin B—β Cyclodextrin System. J. Colloid Interface Sci..

[16] Suárez-Cerda J., Nuñez G.A., Espinoza-Gómez H., Flores-López L.Z. (2014). A comparative study of the effect of α-, β-, and γ-cyclodextrins as stabilizing agents in the synthesis of silver nanoparticles using a green chemistry method. Mater. Sci. Eng. C.

[17] Trindade G.G., Thrivikraman G., Menezes P.P., França C.M., Lima B.S., Carvalho Y.M., Souza E.P., Duarte M.C., Shanmugam S., Quintans-Júnior L.J. (2019). Carvacrol/β-cyclodextrin inclusion complex inhibits cell proliferation and migration of prostate cancer cells. Food Chem. Toxicol..

[18] Ren X., Qian H., Tang P., Tang Y., Liu Y., Pu H., Zhang M., Zhao L., Li H. (2019). Preparation, Characterization, and Properties of Inclusion Complexes of Balofloxacin with Cyclodextrins. AAPS Pharm. Sci. Technol..

[19] Galvão J.G., Cerpe P., Santos D.A., Gonsalves J.K., Santos A.J., Nunes R.K., Lira A.A., Alves P.B., La Corte R.L., Blank A.F. (2019). Lippia gracilis essential oil in β-cyclodextrin inclusion complexes: An environmentally safe formulation to control Aedes aegypti larvae. Pest Manag. Sci..

[20] Sbârcea L., Udrescu L., Ledeţi I., Szabadai Z., Fuliaş A., Sbârcea C. (2016). β-Cyclodextrin inclusion complexes of lisinopril and zofenopril. J. Therm. Anal. Calorim..

[21] Herrera A., Rodríguez F.J., Bruna J.E., Abarca R.L., Galotto M.J., Guarda A., Mascayano C., Sandoval-Yáñez C., Padula M., Felipe F.R.S. (2019). Antifungal and physicochemical properties of inclusion complexes based on β-cyclodextrin and essential oil derivatives. Food Res. Int..

[22] Sancho M.I., Andujar S., Porasso R.D., Enriz R.D. (2016). Theoretical and experimental study of inclusion complexes of β-cyclodextrins with chalcone and 2′, 4′-dihydroxychalcone. J. Phys. Chem. B.

[23] Andrade T.A., Freitas T.S., Araújo F.O., Menezes P.P., Dória G.A.A., Rabelo A.S., Quintans-Júnior L.J., Santos M.R., Bezerra D.P., Serafini M.R. (2017). Physico-chemical characterization and antibacterial activity of inclusion complexes of Hyptis martiusii Benth essential oil in β-cyclodextrin. Biomed. Pharmacother..

[24] Mohandoss S., Atchudan R., Edison T.N.J.I., Mandal T.K., Palanisamy S., You S., Napoleon A.A., Shim J.-J., Lee Y.R. (2019). Enhanced solubility of guanosine by inclusion complexes with cyclodextrin derivatives: Preparation, characterization, and evaluation. Carbohydr. Polym..

[25] de Carvalho L.B., Burusco K.K., Jaime C., Venâncio T., de Carvalho A.F.S., Murgas L.D.S., Pinto L.d.M.A. (2018). Complexes between methyltestosterone and β-cyclodextrin for application in aquaculture production. Carbohydr. Polym..

[26] Ferreira E.B., Da Silva Júnior W.F., De Oliveira Pinheiro J.G., Da Fonseca A.G., Lemos T.M.A.M., De Oliveira Rocha H.A., De Azevedo E.P., Mendonça Junior F.J.B., Neves de Lima Á.A. (2018). Characterization and Antiproliferative Activity of a Novel 2-Aminothiophene Derivative-β-Cyclodextrin Binary System. Molecules.

[27] Quilaqueo M., Millao S., Luzardo-Ocampo I., Campos-Vega R., Acevedo F., Shene C., Rubilar M. (2019). Inclusion of piperine in β-cyclodextrin complexes improves their bioaccessibility and in vitro antioxidant capacity. J. Food Hydrocolloids.

[28] Simsek T., Simsek S., Mayer C., Rasulev B. (2019). Combined computational and experimental study on the inclusion complexes of β-cyclodextrin with selected food phenolic compounds. Struct. Chem..

[29] da Silva Júnior W.F., Bezerra de Menezes D.L., de Oliveira L.C., Koester L.S., Oliveira de Almeida P.D., Lima E.S., de Azevedo E.P., da Veiga Júnior V.F., Neves de Lima Á.A. (2019). Inclusion Complexes of β and HPβ-Cyclodextrin with α, β Amyrin and In Vitro Anti-Inflammatory Activity. Biomolecules.

[30] Bolattin M.B., Nandibewoor S.T., Joshi S.D., Dixit S.R., Chimatadar S.A. (2016). Interaction of hydralazine with human serum albumin and effect of β-cyclodextrin on binding: Insights from spectroscopic and molecular docking techniques. Ind. Eng. Chem. Res..

[31] Nurhidayah E., Ivansyah A., Martoprawiro M., Zulfikar M. (2018). A Molecular docking study to predict enantioseparation of some chiral carboxylic acid derivatives by methyl-β-cyclodextrin. JPhCS.

[32] Saidman E., Chattah A.K., Aragón L., Sancho M., Camí G., Garnero C., Longhi M. (2019). Inclusion complexes of β-cyclodextrin and polymorphs of mebendazole: Physicochemical characterization. Eur. J. Pharm. Sci..

[33] da Câmara Rocha J., da Franca Rodrigues K.A., do Nascimento Néris P.L., da Silva L.V., Almeida F.S., Lima V.S., Peixoto R.F., da Câmara Rocha J., Veras R.C., de Medeiros I.A. (2019). Biological activity of Morita-Baylis-Hillman adduct homodimers in L. infantum and L. amazonensis: Anti-Leishmania activity and cytotoxicity. Parasitol. Res..

[34] Aditya N.P., Vathsala P.G., Vieira V., Murthy R.S., Souto E.B. (2013). Advances in nanomedicines for malaria treatment. Adv. Colloid Interface Sci..

[35] Jambhekar S.S., Breen P. (2016). Cyclodextrins in pharmaceutical formulations I: Structure and physicochemical properties, formation of complexes, and types of complex. Drug Discovery Today.

[36] Cutrone G., Casas-Solvas J.M., Vargas-Berenguel A. (2017). Cyclodextrin-modified inorganic materials for the construction of nanocarriers. Int. J. pharm..

[37] Al-Nasiri G., Cran M.J., Smallridge A.J., Bigger S.W. (2018). Optimisation of β-cyclodextrin inclusion complexes with natural antimicrobial agents: Thymol, carvacrol and linalool. J. Microencapsulation.

[38] Carvalho S.G., Siqueira L.A., Zanini M.S., dos Santos Matos A.P., Quaresma C.H., da Silva L.M., de Andrade S.F., Severi J.A., Villanova J.C.O. (2018). Physicochemical and in vitro biological evaluations of furazolidone-based β-cyclodextrin complexes in Leishmania amazonensis. Res. Vet. Sci..

[39] Oliveira M.G., Brito R.G., Santos P.L., Araujo-Filho H.G., Quintans J.S., Menezes P.P., Serafini M.R., Carvalho Y.M., Silva J.C., Almeida J.R. (2016). α-Terpineol, a monoterpene alcohol, complexed with β-cyclodextrin exerts antihyperalgesic effect in animal model for fibromyalgia aided with docking study. Chem. Biol. Interact..

[40] Trott O., Olson A.J. (2010). AutoDock Vina: Improving the speed and accuracy of docking with a new scoring function, efficient optimization, and multithreading. J. Comput. Chem.

[41] Morris G.M., Huey R., Lindstrom W., Sanner M.F., Belew R.K., Goodsell D.S., Olson A.J. (2009). AutoDock4 and AutoDockTools4: Automated docking with selective receptor flexibility. J. Comput. Chem..

[42] de Sousa Araújo P.S., de Oliveira S.S.C., d′Avila-Levy C.M., dos Santos A.L.S., Branquinha M.H. (2018). Susceptibility of promastigotes and intracellular amastigotes from distinct Leishmania species to the calpain inhibitor MDL28170. Parasitol. Res..

[43] Marinho F.d.A., Gonçalves K.C.d.S., Oliveira S.S.d., Oliveira A.-C.d.S.C.d., Bellio M., d′Avila-Levy C.M., Santos A.L.S.d., Branquinha M.H. (2011). Miltefosine induces programmed cell death in Leishmania amazonensis promastigotes. Memorias Do Inst. Oswaldo Cruz.

[44] Fernandes A.P., Andrade H.M., Melo M.N., Coelho E.A.F., Avelar D., Gazzinelli R.T. (2017). Leishmaniose visceral canina: Novos antígenos para diagnóstico e vacinas. Gerais Rev. De Saúde Públ. Do SUS/MG.

